# New chemistry of transition metal oxyhydrides

**DOI:** 10.1080/14686996.2017.1394776

**Published:** 2017-11-16

**Authors:** Yoji Kobayashi, Olivier Hernandez, Cédric Tassel, Hiroshi Kageyama

**Affiliations:** ^a^ Department of Energy & Hydrocarbon Chemistry, Graduate School of Engineering, Kyoto University, Kyoto, Japan; ^b^ Solid State Chemistry and Materials Group, Institute of Chemical Sciences at Rennes, UMR 6226 CNRS-University of Rennes 1, Rennes, France

**Keywords:** Oxyhydride, hydride, mixed anions, solid-state chemistry, 60 New topics / Others, 107 Glass and ceramic materials, 201 Electronics / Semiconductor / TCOs, 203 Magnetics / Spintronics / Superconductors

## Abstract

In this review we describe recent advances in transition metal oxyhydride chemistry obtained by topochemical routes, such as low temperature reduction with metal hydrides, or high-pressure solid-state reactions. Besides the crystal chemistry, magnetic and transport properties of the bulk powder and epitaxial thin film samples, the remarkable lability of the hydride anion is particularly highlighted as a new strategy to discover unprecedented mixed anion materials.

## Graphical abstract

**Figure:**
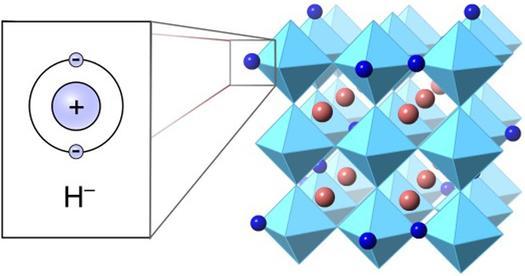

## 1. Introduction

In the early 1990s, the chemistry of metal oxyhydrides had been the subject of very scarce studies, mostly implicating electropositive *p*-group elements (eventually combined with transition metals to form an alloy) or more seldom intermetallic compounds containing non-metallic elements. Despite their very similar ionic radii (*ca.* 1.4 Å), the coexistence of hard oxide anions and soft, polarizable, hydride anions with a lower charge in a crystalline solid was usually considered as thermodynamically unlikely except under very reducing conditions able to stabilize the hydride anion [1].

The discovery of the very first mixed oxyhydride solid is considered as dating from 1982 with the solid-state synthesis at 900 °C under pure hydrogen of the ternary compound LaHO [2]. Subsequent X-ray and neutron powder diffraction experiments [3] pointed out that this material crystallizes in a fluorite superstructure featuring an ordered cubic anionic environment for lanthanum characterized by hitherto unseen oxygen–hydrogen contact distances of 2.85 Å. Such distances are specific of negatively charged hydrogen species, as they are much longer than the O^2–^–H^+^ interatomic distance encountered in OH^–^ groups ranging from 1.32 to 1.37 Å. It is worth pointing out that LaHO turned out to be readily hydrolysable under ambient moisture, thereby releasing hydrogen. Later on, only few other stable materials containing both oxide and significant amount of hydride anions were reported, such as oxygen-stabilized η-carbides Zr_3_V_3_OD_*x*_ (*x*_max_ = 4.93; D^–^ within Zr and V-tetrahedral interstices) [4] or Zr_5_Al_3_O_1–*x*_, an oxygen-stabilized Nowotny phase able to uptake up to 4.8 interstitial hydride per formula unit [5]. One can also cite the inverse perovskite-type Ba_3_AlO_4_H [6] or the hydrogen-stabilized Zintl phase of barium Ba_21_T_2_O_5_H_*x*_ (T = Ge, Si, Ga, In, Tl; *x*_max_ = 24) [7] synthesized under drastically reducing conditions (1100 °C under a H_2_ pressure of ~1 bar). In both latter compounds, isolated hydride occupies interstices inside distorted barium polyhedra.

Regarding more specifically transition metal oxyhydrides and anion substitution, thermodynamics suggest that only oxides with a strong formation enthalpy are prone to survive under reducing conditions and hence form oxyhydrides. Second, it should be recalled that the hydride itself is a very reducing chemical species, able to reduce the transition metal cation to the metal. Nonetheless, using a soft chemistry method (‘chimie douce’), Rosseinsky et al. reported in 2002 the low temperature CaH_2_ solid-state synthesis of LaSrCoO_3_H_0.7_ [8]. Contrary to the aforementioned examples of electropositive main group metal oxyhydrides, the chemical route at play here is of topochemical type—keeping intact the architecture of the layered perovskite reactants—and moreover in LaSrCoO_3_H_0.7_ cobalt–anion–cobalt pathways for magnetic exchange interactions are present. Interestingly, those pathways exhibit different strengths depending on whether an oxide or a hydride is locally present, allowing control of the electronic or magnetic properties. This new layered perovskite cobalt oxyhydride and its successors *Ln*SrCoO_3+*a*_H_*b*_ with *Ln* = Pr, Nd [9] and Sr_3_Co_2_O_4.33_H_0.84_ [10] exhibit a rather low formal oxidation state for cobalt +1.7 and +1.75, respectively, that was considered as a stabilization condition for hydride-oxide systems [11]. The discovery in 2012 by Kobayashi et al. of an unanticipated Ti^3+/4+^ barium oxyhydride, exhibiting hydride exchange and electronic conductivity [12], has renewed the chemistry of transition metal oxyhydrides. Here, the oxidation state of the *B*-site cation is not unusual, as many other Ti^3+^ compounds exist. Ever since, this family of materials has steadily grown, based on not only the topochemical route but also with the use of high-pressure solid-state reactions, extending the compositions to the Sc [13], V [14,15], Cr [16] or Mn [17] elements on top of the initial Co or Ti oxyhydrides.

The aim of this review is to present this new family of transition metal oxyhydrides, obtained by low temperature reduction with metal hydrides, or under high pressure and high temperature. Additionally, the synthesis, crystal chemistry, physical properties of the bulk powder and epitaxial thin film samples, and the remarkable lability of the hydride anion as a new strategy to discover unprecedented mixed anion materials will be presented.

## 2. Synthesis of oxyhydrides

The synthesis of oxyhydrides requires special care, given the unusual properties of the hydride anion. Preparation typically requires strongly reducing conditions, so excessive reduction of 3*d* cations to the metallic state may occur. Additionally, at high temperatures, many known hydride compounds decompose via H_2_ release, so this is a potential issue in any oxyhydride compound. Treating oxides with H_2_ typically only reduces the oxide to the metallic state at most; so far no syntheses of transition metal oxyhydrides from H_2_ gas have been reported yet. Hence, the use of a hydride itself as a starting material is necessary; potential examples are the use metal hydrides such as CaH_2_, NaH, and TiH_2_, which may be commercially obtained or prepared in the lab by treating metals with H_2_ gas at moderate temperatures. These starting materials are naturally useful in the synthesis of various Ca, Na, and Ti-containing transition metal oxyhydrides, but one is not bound to these compositions. For example, CaH_2_ can be reacted with another oxide, and the resulting CaO can be removed from the reaction mixture by washing. These typically involve topochemical reactions. Hence, there are two main approaches to preparing oxyhydrides: the first involves the topochemical reduction of a parent oxide followed by removal of unwanted byproducts; the second involves a more direct synthesis from a mixture of hydride and oxide precursors, with no removal of intended byproducts. Recently, the latter has often been conducted under high pressure.

### 2.1. Topochemical synthesis

In solid-state chemistry, a topochemical synthesis is a synthesis based on the conversion of a parent compound converting to a product based on a very limited rearrangement of atoms, where the only bonds broken/formed are those which are necessary for the transformation. Topotactic reactions, a related but not necessarily exclusive term, signify that the overall symmetry of the structure before and after reaction are closely related. One simple example is ion exchange, whereby select atoms are exchanged while the other atoms do not diffuse and rather preserve the original crystal structure. Thus, unlike most high-temperature solid-state reactions, the structures of the starting materials and products of topochemical synthesis resemble each other quite closely. As low temperatures are involved and only ‘minimal’ changes to the structure are made, metastable products may also result. In terms of removing O^2–^ (for subsequent replacement with H^–^) from oxides, the early work of Rosseinsky and Hayward [18–21], Kageyama [22–26], and Greenblatt [27] provides a starting point. For example, the three-dimensional (3D) perovskite LaNiO_3_ can be treated with NaH at relatively low temperatures (200–300 °C) to yield the layered LaNiO_2_ structure and NaOH/Na_2_O, which is washed away [18]. The resulting LaNiO_2_ structure resembles the original perovskite lattice, only in that selected ‘apical’ oxide anions have been removed. Similar novel oxygen-deficient structures have been reported for Mn [20,28], Fe [22–24] and Co [19,21]. Of course, these are not oxyhydrides, but the procedure for preparing oxyhydrides remains essentially the same.

**Figure 1.**
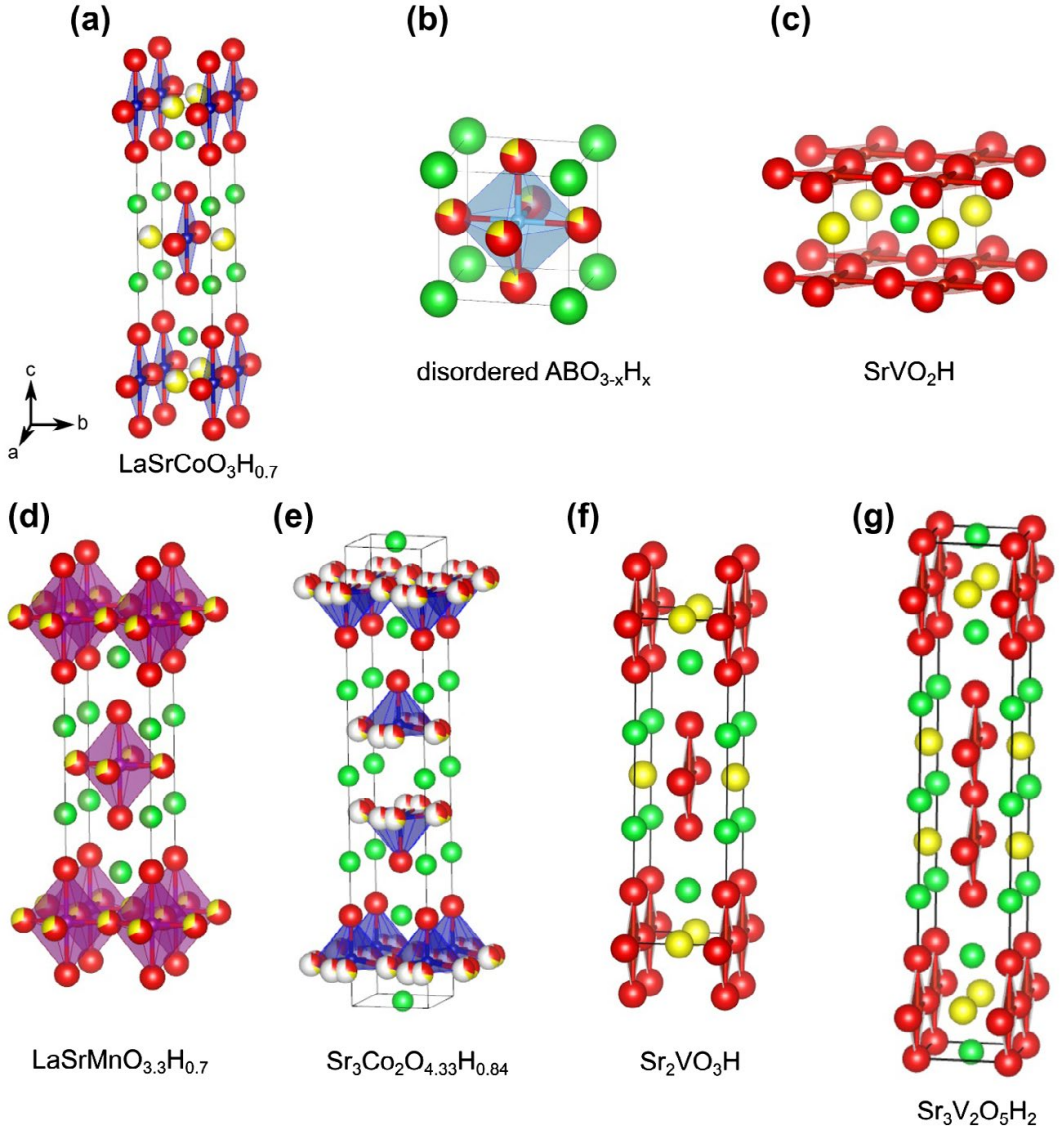
Structures of various oxyhydride compounds. For all structures, unit cells are oriented in the same *a*, *b*, *c* direction, as indicated in (a). Hydride is shown by yellow spheres, and oxide by red spheres. For the layered structures, interlayer La and Sr cations are shown by shades of green. For disordered ABO_3–*x*_H_*x*_ in (b), the primitive cubic cell seen with BaScO_2_H, SrCrO_2_H, SrTiO_3–*x*_H_*x*_, and BaTiO_3–*x*_H_*x*_ is shown; octahedral tilting is observed for certain Ca-Sr solid solutions (Ca,Sr)TiO_3–*x*_H_*x*_, resulting in larger unit cells (not shown).

The first transition metal oxyhydride, LaSrCoO_3_H_0.7_ (see Figure 1(a)) was prepared by the topochemical reduction of LaSrCoO_4_ [8]. Other subsequent oxyhydrides which have been reported are titanates, such as (Ba, Sr, Ca, Eu)TiO_3–*x*_H_*x*_ [12,29,30] (structure shown in Figure 1(b)), and vanadates, such as SrVO_2_H [15] (Figure 1(c)). All of these examples involve grinding the parent oxide with CaH_2_ and pelletizing within a glovebox, followed by sealing in an evacuated glass ampoule. Heating to 300–600 °C results in the oxyhydride product and CaO by-product, the latter of which is washed away in a methanol solution of weak acid (ammonium chloride). This method has also been applied to the preparation of oxyhydrides in thin form. Typically the parent oxide (SrTiO_3_, LaSrCoO_4_, etc.) is deposited on a suitable substrate via vacuum techniques such as pulsed-laser deposition. The film/substrate is then treated in the same way as powder, by immersion in loose CaH_2_ powder and sealing in a Pyrex tube under vacuum. Consequent heat treatments can be conducted at slightly lower temperatures. In this way, films of (Ba, Ca,Sr)TiO_3–*x*_H_*x*_ [31,32] and LaSrCoO_4−*x*_H_*x*_ [33] have been prepared.

While not being a transition metal oxyhydride, we note that the interesting calcium aluminate oxyhydride C12A7:H^–^ is also formed from its parent oxide (C12A7 for mayenite 12CaO ⋅ 7Al_2_O_3_) either by reaction at 1300 °C with 20% H_2_/80% N_2_ [34] or alternatively using a CaH_2_ reduction process at 800 °C leading typically to a higher hydride content than the high-temperature solid–gas route [35]. For the titanate cases, the amount of hydride can be varied within *x* = 0–0.6 for a BaTiO_3–*x*_H_*x*_ formula by adjusting the temperature and CaH_2_ amount. However, increasingly strenuous conditions eventually lead to decomposition of the original oxide framework, observed in terms of reduced crystallinity and increasing amounts of TiH_2_ [12]. We further note that it is not yet possible to predict whether an oxygen-deficient product or oxyhydride product will result; for example, an anatase TiO_2_ film simply gives a dark-colored TiO_2–*x*_ phase when treated with CaH_2_ [36], whereas BaTiO_3_ gives the oxyhydride. La_2_Ti_2_O_7_ contains only minute amounts of hydride [37].

Most hydride reductions to date have used CaH_2_, with fewer examples with NaH and LiH. We can see that the surrounding thermodynamics differ when using these various hydrides [38]. For example, in the absence of any oxide to reduce, NaH and CaH_2_ will decompose at high temperatures:

(1) NaHs→Nal+12H2g

(2) $${\text{CaH}}_{\text{2}}\left(\text{s}\right)\to \text{Ca}\left(\text{s}\right){\;\text{+ H}}_{\text{2}}\left(\text{g}\right)$$

As Ca^2+^ is a divalent cation, the lattice enthalpy of CaH_2_ is considerably higher than that of NaH. Thus, while at 210 °C a H_2_ pressure of 10 Torr may result, the equilibrium constant for eq. 2 is predicted to be only 10^−8^ even at 400 °C (assuming Δ*H*° and Δ*S*° values are independent of temperature). Hence, CaH_2_ is probably suitable at a higher temperature rather than NaH, otherwise, the experiment simply becomes a reduction using pure Na or Ca metal. Obviously, a suitable reduction temperature also depends on how easily the target oxide can be reduced (e.g. Fe vs. Ti), but the trend above still holds.

In comparing these hydride reducing agents, it is also necessary to compare the thermodynamics of the by-products. For example, a reduction with NaH could possibly yield either Na_2_O or NaOH; similarly, CaH_2_ could yield either Ca(OH)_2_ or CaO. The question in essence here is whether a one-electron or two-electron reduction is involved [38], that is,

(3) $${\text{H}}^{-}\to {\text{H}}^{\;\text{+}\;}{\;\text{+ 2e}}^{-}$$

(4) $${\text{H}}^{-}\to \text{1}\;/\;\text{2}{\text{H}}_{\text{2}}{\;\text{+ e}}^{-}$$

In reality, NaOH is quite stable compared to Na_2_O, as demonstrated for example by the extreme difficulty of dehydrating NaOH to Na_2_O. Hence, reductions with NaH probably result in the two-electron reduction, yielding NaOH rather than Na_2_O. Another further point to consider is the side reaction between any hydroxide by-product and remaining hydride:

(5) $$\text{NaH + NaOH}\;⇄{\;\text{Na}}_{\text{2}}{\text{O + H}}_{\text{2}}\left({\text{K}}_{\text{eq},400^{\circ }\text{C}}{\;\text{= 10}}^{-6}\right)$$

(6) $$\text{Ca}{\left(\text{OH}\right)}_{\text{2}}{\;\text{+ CaH}}_{\text{2}}⇄{\;\text{2CaO + 2H}}_{\text{2}}\left({\text{K}}_{\text{eq},400^{\circ }\text{C}}{=10}^{12}\right)$$

For NaH, the small *K*_eq_ of Equation (5), combined with the dominance of Equation (3) over Equation (4) imply that any hydrogen pressure during the experiment is the result of a thermal decomposition (Equation (1)), rather than reduction of the parent oxide. This should be taken into account during synthesis, as the experiment may be more sensitive to reaction temperature and ampoule volume.

In the case of CaH_2_, only a small amount of H_2_ will be released from thermal decomposition. Rather, H_2_ will be released from either one-electron reduction of the oxide (Equation (4)), or any Ca(OH)_2_ impurities with the CaH_2_. Depending on the reactant quantities, this pressure can be substantial (up to 20 atm), leading to minor explosions. The effect of H_2_ pressure on the oxyhydride formation has been briefly discussed previously [9], and should be taken into account when standardizing experimental procedures. Gas-phase contributions toward the formation of oxygen-deficient oxides using CaH_2_ and NaH have been demonstrated in the past [18,38].

The benefits of these topochemical hydride synthesis are the relative ease (compared with the next method) in terms of equipment. Additionally, synthetic explorations are simple to plan, where one may choose any (in theory) oxide where one believes that there is O^2–^ which may be removed/exchanged, and the products typically do not yield multiple phases or drastically different crystal structures, making structural analysis straightforward. The drawback, however, lies in the inherent *reduction* nature of the process itself; that is, for the reaction to occur, the transition metal must undergo a reduction. For example, BaTiO_3_ (Ti^4+^) is reduced to BaTiO_2.5_H_0.5_ (Ti^3.5+^). Zr, however, is considerably more difficult to reduce, and the synthesis of BaZrO_2.5_H_0.5_, or other irreducible elements such as Sc or Al is difficult to conceive. Hence, while there are still no Nb, Ta, or W oxyhydrides reported so far, for example, the explorative space is eventually limited by the repertoire of parent oxide. Additionally, over-reduction can also occur; our attempts to prepare iron-based oxyhydrides almost always result in the formation of Fe metal.

### 2.2. Direct synthesis via high pressure

**Figure 2.**
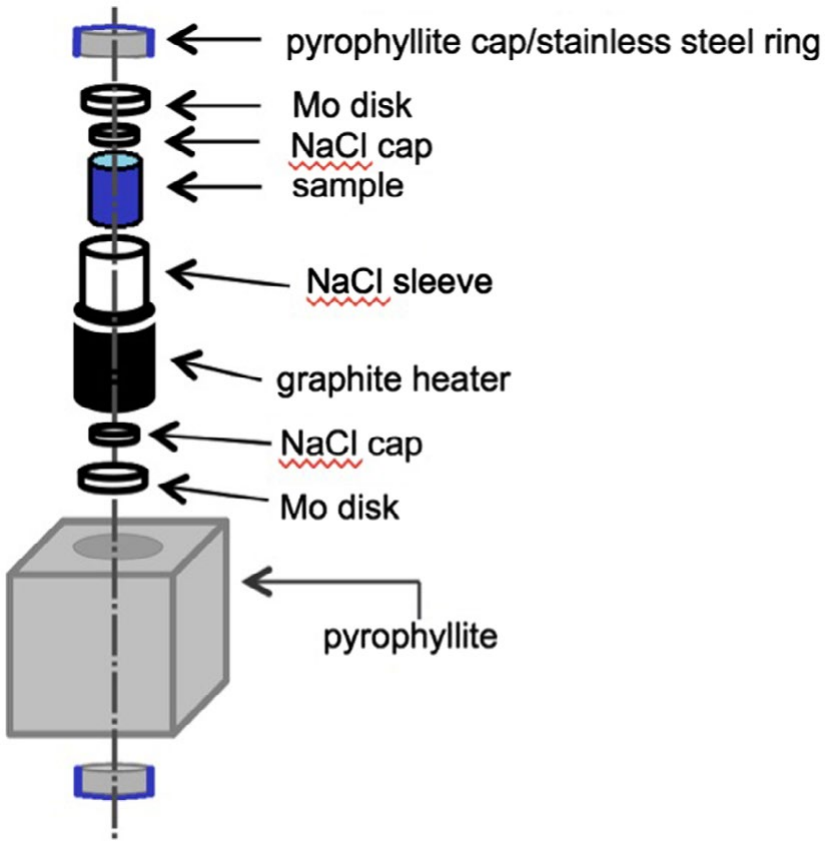
Schematic of a sample cell for high-pressure of oxyhydrides. The pyrophyllite cube is approximately 1.5 cm × 1.5 cm × 1.5 cm. Tungsten carbide blocks (anvils) apply pressure to the pyrophyllite cube from all six faces. Heating is achieved by electric current, via electrical contacts running through the tungsten carbide anvil, steel ring, and Mo disk to the graphite heater.

A direct synthesis of transition metal oxyhydrides without any redox processes eliminates the problems encountered above for topochemical reductions. Hypothetically, BaTiO_2_H could be synthesized by reacting stoichiometric amounts of BaH_2_, BaO, and Ti_2_O_3_. The method can be used to achieve compositions based on irreducible elements, such as SrAlO_2_H. However, BaH_2_ and other simple hydrides often decompose to release H_2_ gas at the elevated temperatures necessary to achieve these solid-state reactions. The use of solid-state high-pressure techniques eliminates this problem by enclosing the reaction mixture in a solid container with virtually no head space; heating under these conditions prevents the loss of hydrogen, making oxyhydride synthesis possible. A sample cell is shown in Figure 2. To form a gas-tight seal, care must be taken in choosing the cell materials. In our own research, we have found that Au and Pt components seem to react with alkali/alkali earth reagents. NaCl sleeves work well, as they are soft enough to fuse into gas-tight seals under pressure, unlike other materials such as BN or Pt foil. NaCl has the additional advantage in that it may be removed by washing. Supplementary sources of H_2_ can be included within the cell, as with a mixture of NaBH_4_/Ca(OH)_2_ [14]. The high-pressure technique has been quite successful in forming the oxyhydride compounds; to date Sr_2_VO_3_H [14], SrCrO_2_H [16], LaSrMnO_3.3_H_0.7_ [17], and BaScO_2_H [13] have been reported (see Figure 1).

## 3. Specificity of oxyhydride characterization

Generally speaking, hydride as a negatively charged hydrogen atom should be characterized as any hydrogen species in a solid. The difficulty to chemically and structurally characterize transition metal oxyhydrides depends obviously on the level of hydride content in the investigated material. For oxyhydrides prepared by direct synthesis, an initial estimation of the hydride content can be made based on the reaction stoichiometry, but for topochemical reactions the amount of hydride will depend on the temperature and duration of the CaH_2_ reduction step. Furthermore, at the crystal-averaged atomistic level, the location of hydride anions in the unit cell may be difficult to identify owing to the ordered or disordered character of the anionic sublattice. In the latter case, the presence of H^–^ on an anionic site also statistically occupied by an oxide anion is not so straightforward to ascertain and it is quite easy to miss. In fact these three species, hydride, oxide and vacancies, can in principle be present in the material and thus can coexist on the same crystallographic site, constituting a complex situation. Moreover, the simultaneous presence of hydroxide molecules coexisting with hydride anions is also possible in metal oxides or calcium phosphates as brought up recently by Hayashi et al. by nuclear magnetic resonance (NMR) [39] or based on maximum entropy method (MEM) analysis of neutron diffraction data by Masuda et al. [40]. The combined use of different characterization techniques is therefore highly required. The sought chemical composition is actually dependent on several assumptions, for instance, the correctness of the mass fractions refined by Rietveld analysis in case of coexisting phases and the oxygen stoichiometry of the eventually remaining starting pure oxide. These highlight the difficulty to get an unambiguous chemical and structural description of highly disordered oxyhydrides. We summarize below most of the techniques used so far for characterizing oxyhydrides, underlining their respective advantages and disadvantages.

Diffraction techniques, namely laboratory or synchrotron X-ray powder diffraction (XRD) as well as neutron powder diffraction (NPD), are widely used to investigate the structure and composition of obtained products. Clearly NPD, although not always accessible within a reasonable period of time, is required for a correct structural analysis of oxyhydrides as XRD is not able to detect hydrogen in the presence of heavy elements (e.g. Sr, Ba, La, Pr, Nd etc.). For Eu [30,41], or other highly absorbing elements (e.g. Sm, Gd, Dy) NPD should be nonetheless avoided, though not impossible with a special cell arrangement [41]. High-resolution time-of-flight (TOF) NPD instruments are often more suited than constant-wavelength diffractometers to determine subtle structural features, for instance coexistence of O^2–^ and H^–^ on the same site, as shown in *Ln*SrCo_3+*α*_H_*β*_ oxyhydrides (*Ln* = Pr, Nd) [9]. A combined Rietveld refinement of XRD and NPD data collected at the same temperature allows a structural model more robust and less prone to least-squares correlations between occupancy and atomic displacement parameters, as they help in benefitting from very different atomic scattering factors of each chemical species with X-rays and neutrons. Such an approach is particularly useful to demonstrate the hydride location and the chemical occupancies of each site [8,12]. The eventual presence of impurity phases plus small amount of starting material may make the multi-phase Rietveld refinement challenging. It is worth noticing it is not always possible to exclude by diffraction the possible presence of a low amount of oxide anions on a hydride site. If resulting metal-O bond length is rather short (e.g. 1.80 Å vs. 2.18 and 1.94 Å for the pure oxide O1-apical and O2-equatorial sites in LaSrCoO_3_H_0.7_ [8]) this assumption can be reasonably discarded, by relying also on chemical analysis results.

In the course of the structural determination of LaSrCoO_3_H_0.7_ [8], a pure oxide, without hydride anion, was first proposed from synchrotron XRD data according to an *Immm* Sr_2_CuO_3_-type model consisting of chains of corner-sharing CoO_4_ squares. Intuitively, it would correspond to a reductive topotactic transformation from LaSrCo^+III^O_4_ (octahedral cobalt) into the LaSrCo^+I^O_3_ structure (square planar cobalt) during which the oxide anions which connect the octahedra in the *b* axis within the equatorial plane were removed in an ordered manner (see Figure 1(a)). While NPD gave a satisfactory fit for magnetic reflections, the nuclear peaks remained still poorly modeled by the *Immm* LaSrCoO_3_ structural model. A difference Fourier map showed the presence of a substantial hole of negative nuclear density at these sites. As hydrogen is one of five natural elements to exhibit a negative coherent scattering length (together with Li, V, Ti and Mn), it was successfully incorporated in the model at this position, yielding in a greatly improved refinement.

In a similar manner, for BaTiO_3–*x*_H_*y*_ [12] it was the significant discrepancy between the oxygen content refined from the synchrotron XRD data and from the NPD data on the other hand that led to suspect the presence of hydride. Initially, after reduction of BaTiO_3_, a refined stoichiometry of BaTiO_2.59(6)_ was obtained from synchrotron XRD data. An independent refinement of the NPD data led to a much lower oxygen content (BaTiO_1.91(3)_), corresponding to an unrealistic Ti valence of +1.82. Such discrepancy in terms of oxygen content derived from NPD and XRD diffraction experiments could be explained only by the presence of negative-scattering hydrogen randomly disordered with oxide on the single anionic site, decreasing the apparent nuclear density of this site with respect to the initially calculated density. If only oxygen is considered by NPD Rietveld (ignoring hydrogen) its content is then underestimated, while by XRD—insensitive to the presence of hydrogen—the oxygen content is correctly determined, although intrinsically less accurate as oxygen is a light element for X-rays in presence of heavy elements. Introducing hydrogen on the anionic site and refining again the NPD data alone increased the oxygen content to BaTiO_2.33(2)_, a value much closer to the one obtained by synchrotron XRD. A combined Rietveld refinement of XRD and NPD data was conducted to obtain the final refined formula, BaTiO_2.38(1)_H_0.62_. NPD allowed additionally to discard the presence of hydroxide molecules in the solid as Fourier difference maps did not show any significant negative holes at around 1 Å from the anionic site. Based on the agreement of X-ray and neutron refinement data with a model consisting of only hydride and oxide at the anionic site, the combination of these two data-sets also revealed the total or almost total absence of vacancies.

**Figure 3.**
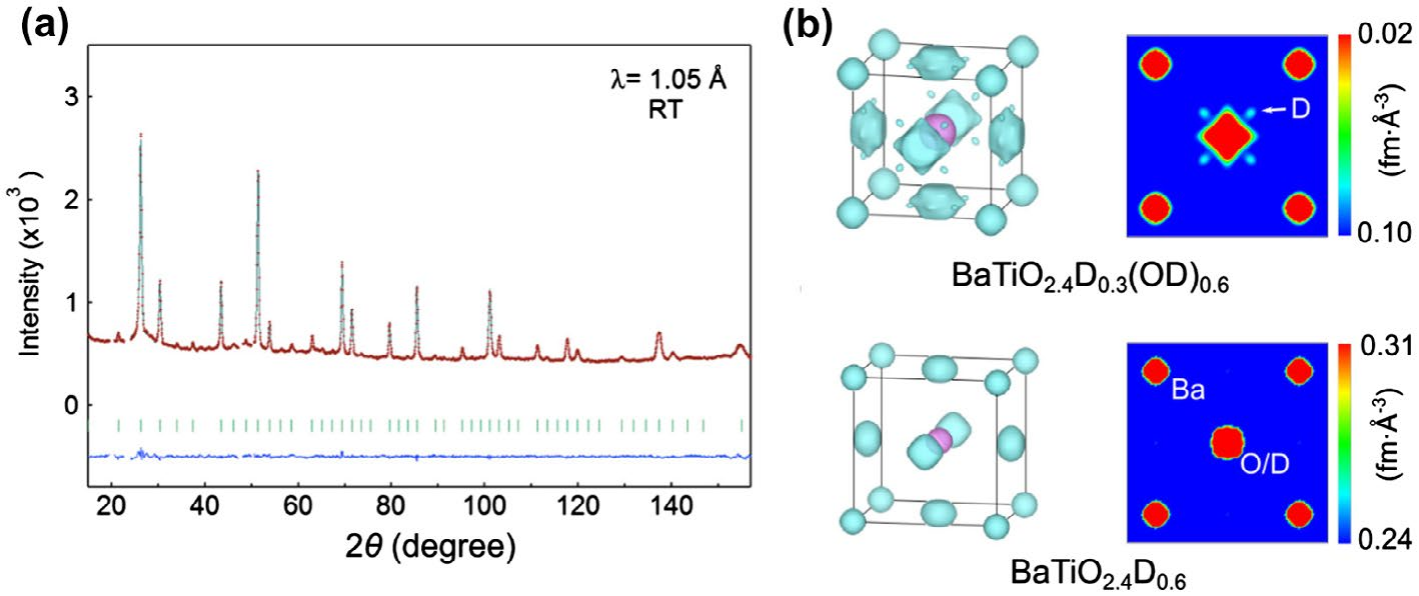
Neutron diffraction pattern (a) and nuclear density maps from MEM analysis (b) to differentiate anionic species.

The MEM applied to powder neutron diffraction data [42]) is in principle more accurate than conventional Fourier difference maps, leading to less biased nuclear density maps with less background. Figure 3(a) shows a neutron diffraction pattern of a BaTi(O^2–^, D^–^, OD^–^), and a nuclear density map based on MEM analysis in Figure 3(b). Small spots of nuclear density are observed approximately 1 Å from the oxygen centers, signifying the presence of hydroxide (in deuterium form). In contrast, MEM analysis of BaTiO_2.4_D_0.6_ does not exhibit any such lobes (Figure 3(c)). Thus, this method was able to suggest the stable coexistence of H^+^ and H^–^ at ambient conditions in BaTiO_2.4_(D^–^)_0.3_(OD^–^)_0.3_ [40], and a subsequent combined Rietveld refinement of neutron and X-ray diffraction data was used to obtain the precise anionic stoichiometry.

Other techniques than diffraction are used to qualitatively and quantitatively determine the hydride content in oxyhydrides. For example, mass spectrometry (as typically encountered in a TG-MS setup) can be used. The presence of hydride in LaSrCoO_3_H_0.7_ [8] was chemically confirmed by quantitative mass spectrometric monitoring of the H_2_O evolved simultaneously with oxidation of the oxyhydride material under flowing O_2_(g) at 272 °C, yielding 0.4 H^–^ per formula unit, a value almost half of the hydride stoichiometry determined by diffraction. Of course, heating hydroxides also yields H_2_O, so the release of H_2_ when heating under inert atmospheres such as Ar or He can be taken as an indication of hydride within the lattice [12]. Quantification of hydride is not trivial, but may be accomplished with rigorous use of internal standards within the gas stream and standard hydride samples. Another approach to quantification has been acid digestion of the sample, and volumetric/mass spectrometric analysis of the evolved gases. For example Yoshizumi et al. and Kobayashi et al. dissolved their C12A7:H^–^ [43] and BaTiO_3–*x*_H_*x*_ samples [12] in DCl/D_2_O or D_2_SO_4_, and collected the evolved gases to prove and quantify the existence of lattice hydride.

As a related technique to heating the sample under inert atmospheres and measuring the released H_2_ gas, thermal desorption spectroscopy [44] has also been used as a characterization tool. This typically requires a dedicated instrument, composed of a high vacuum chamber, in-line mass spectrometer, and IR heating stage. Quantification is possible with careful calibration, and was used by Bouilly et al. [33] and Bang et al. [14] for their thin film and powder samples.

Thermogravimetry analysis (TGA) can be used under oxidative atmosphere to confirm the suspected stoichiometry via weight change. Any oxyhydride obtained by topochemical reduction can be reoxidized to the original parent oxide, undergoing a weight change reflecting the original hydride content, assuming there are no anion vacancies. For BaTiO_3–*x*_H_*y*_ [12] the refined formula from Rietveld analysis, BaTiO_2.38(1)_H_0.62_, compares nicely well with the one deduced from TGA in air, BaTiO_2.38_H_0.62_. Reductive TGA may also be performed; for Sr_3_Co_2_O_7–*y*_H_*y*_ [10] the observed relative mass loss by reductive TGA under 5%H_2_ in N_2_ was used (in combination with other results) to confirm a Sr_3_Co_2_O_4.33_H_0.84_ formula.

TGA experiments revealed in *Ln*SrCo_3+*α*_H_*β*_ oxyhydrides (*Ln* = Pr, Nd) an oxygen stoichiometry substantially higher than 3.0 (up to 3.33) [9], contrary to LaSrCoO_3_H_0.70_. Based on this additional information, extra oxide anions were introduced in the crystallographic model on or very close to the hydride site bridging the cations in the *ab* plane. This implied that the substitution of oxide for hydride within the CoOH_1–*x*_ sheet was incomplete in those Pr- and Nd-containing cobalt oxyhydrides; the additional substitutional disorder in the transition metal layer induced local variations in the Co environment such as displacements of the pure oxide O2 equatorial site handled by a ‘split-atom’ model.

Simple, routine elemental analysis using commercial CHN analyzers can play an important role to quantitatively confirm the results from NPD/synchrotron XRD combined Rietveld refinement. Regarding LaSrCoO_3_H_0.7_ [8] a more accurate global hydrogen content was obtained by elemental analysis, revealing 0.26(2)% H by mass, very close to the 0.21% mass from the combined Rietveld refinement, definitely confirming the absence of oxide anion on the hydride site, which is statistically occupied by only hydrides and vacancies. ^1^H magic angle spinning nuclear magnetic resonance spectroscopy (MAS NMR) qualitatively confirmed the presence of hydride in the Sr_3_Co_2_O_7_ majority phase by comparison with a CaH_2_ spectrum [10]. ^1^H MAS NMR measurements otherwise confirmed the same chemical state and environment for hydrogen species in BaTiO_3–*x*_H_*y*_ [12]. As mentioned above, H^–^/OH^–^ are not so simple to differentiate by NMR (see the recent study by Hayashi et al. [39]). But this spectroscopic technique remains one of the few tools for exclusively seeing hydrogen and its position, though not so usable if one deals with magnetic or conducting samples.

Use of H/D isotopes can help when dealing with neutron diffraction or mass spectrometry. The H^–^ and D^–^ exchange within BaTiO_3–*x*_H_*y*_ was shown to occur in D_2_(g) atmosphere at 400 °C monitored *in situ* by MS, confirmed by NPD experiments before and after deuteration [12].

The valence of the transition metal can be checked by Curie–Weiss fitting against magnetic susceptibility data, on the other hand X-ray absorption spectroscopy (XAS) is also used to confirm the formula. The average oxidation state of Co calculated from the refined anionic content in LaSrCoO_3_H_0.7_ [8] was shown to be consistent with an X-ray absorption Co *K*-edge position very close to that of the Co(II) standard (LaSrCoO_3.5_). In BaTiO_3–*x*_H_*y*_ magnetic susceptibility data showed a +3.4 oxidation state for Ti strengthening more the refined formula [12].

Muon spin rotation (μSR) experiments confirmed LaSrCoO_3_H_0.7_ was magnetically ordered [8] and quasi-elastic neutron scattering experiments [45] were carried out to probe dynamics of the same material within the –0.4 and 0.4 meV energy transfer window with an instrumental resolution of 17.5 meV in FWHM. This corresponds to a timescale of 1–75 ps. The onset of hydride mobility along the *a* axis within the perovskite layer was revealed above 402 °C, associated with an ionic conductivity of *ca.* 3–5 S cm^−1^ around 427 °C, significantly higher than in protonic conductors.

Besides the different relevant techniques mentioned hereinabove (XRD, NPD, TGA, MS, magnetic susceptibility, NMR, XAS, chemical analyses etc.), hydrogen forward scattering (HFS) [46] can also be cited as another experimental method used so far for the analysis of TM oxyhydrides [30], quantification requiring curve fitting.

As far as they are concerned, epitaxial thin films of *A*TiO_3–*x*_H_*x*_ (*A* = Ba, Sr, Ca) [31] were characterized by XRD and by secondary ion mass spectroscopy (SIMS). Low temperature CaH_2_ reduction and subsequent hydride insertion was shown to induce small change in unit cell parameters relative to the as-grown oxide film. SIMS, that is an expensive technique and requires standards for quantification but compulsory for thin films, gave hydrogen density (that turned out to be large, e.g. SrTiO_2.75_H_0.25_) and its depth dependence, pointing out a uniform distribution in the film.

## 4. Chemical reactivity of oxyhydrides: lability and exchange

The chemical reactivity of hydride is unlike that of any other anion. The redox potential of H^–^ between H_2_ and H^–^ is highly negative (–2.23 V). Thus, H^–^ can be readily oxidized to H_2_ gas. The oxidant may be an external species, or one of the metal cations within the compound itself; the latter is more conveniently described as a reductive elimination of hydride. This property is illustrated in the release of H_2_ from TiH_2_ when heated at approximately 400 °C, for example [47,48].

**Figure 4.**
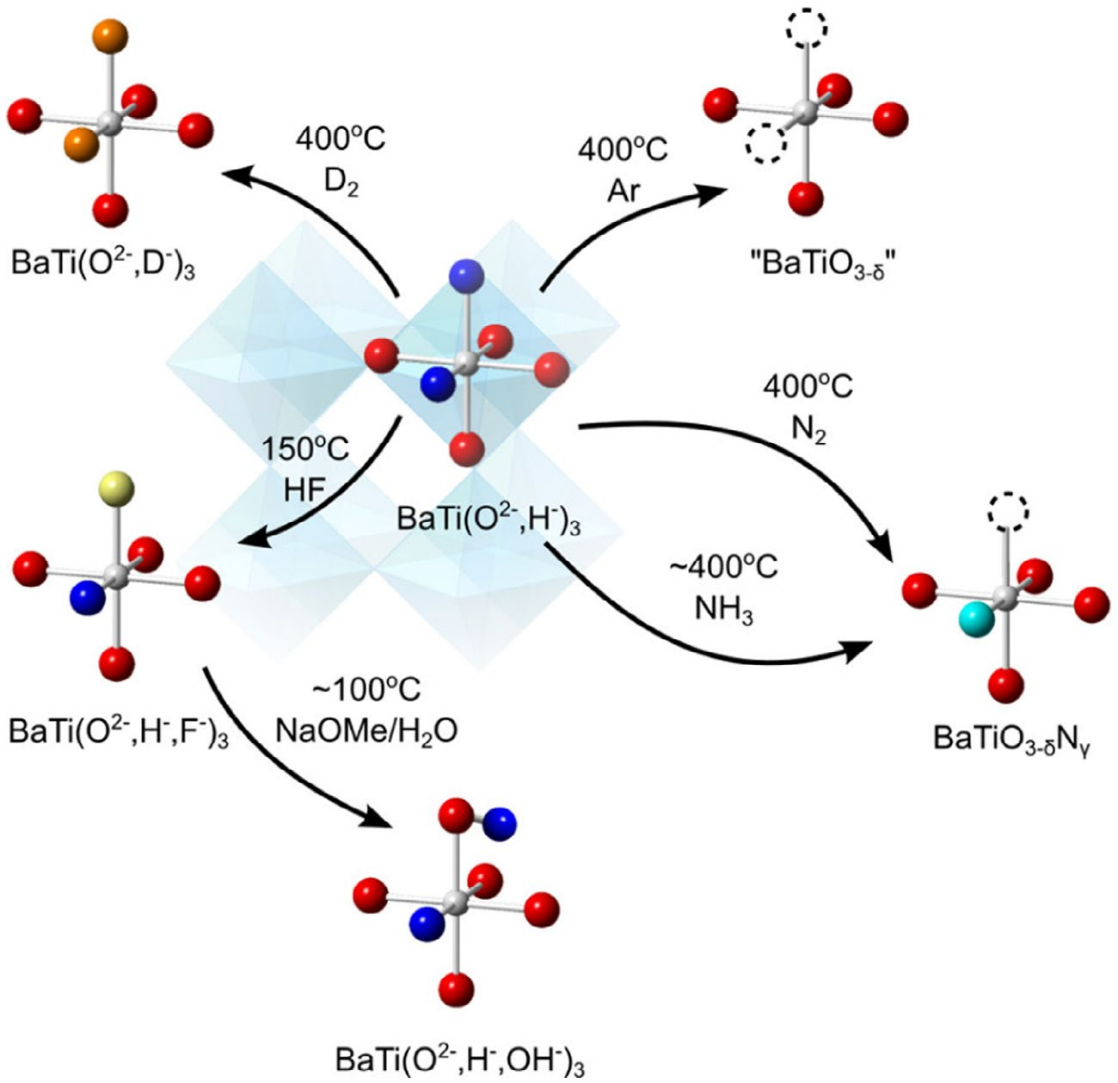
Different anion exchange routes starting from an oxyhydride.

This release of H_2_ can also be seen in certain oxyhydrides, and in reversible manner. Using a TG-MS apparatus under flowing Ar, Kobayashi et al. observed the release of H_2_ from BaTiO_2.4_H_0.6_ at approximately 400 °C during a linear temperature ramp from room temperature to 600 °C (and presumably yielding a BaTiO_3–*δ*_ product, see Figure 4). Repeating the experiment under D_2_ gas led to the detection of HD gas, also peaking at 400 °C. The oxyhydride product was found to be almost fully deuterated (based on neutron diffraction), indicating that hydride exchange with gaseous hydrogen is possible at 400 °C, the same temperature as the release temperature.

This demonstrated thermolability of hydride is quite interesting as it makes anion exchange possible. In solution chemistry, the broad range of various inorganic complexes rests upon the fact that various (precursor) ligands are *labile*, that is, they do not bind too strongly neither too weakly, making consequent ligand exchange possible. Hydride in oxyhydrides appears to be of similar use. Other than the thermal lability described above, we note that H^–^, as a base, would also be prone to acid-base reactions with acids, also permitting it to leave as H_2_ gas under moderate conditions.

The thermal lability for synthetic purposes has been demonstrated by Yajima et al. [49] and Masuda et al. [40] recently by their synthesis of oxynitride BaTiO_3–*x*_N_*y*_. Typically, the preparation of oxynitrides has required the use of NH_3_ at high temperatures of 800–1300 °C. In the case of preparing the oxynitride version of BaTiO_3_, the highest nitrogen content has been BaTiO_2.85–*δ*_N_0.1_ which was prepared with flowing NH_3_ at 950 °C. In contrast, by the use of a BaTiO_2.4_H_0.6_ precursor, treatment with NH_3_ at temperatures of 375–550 °C led to considerably higher nitrogen contents of BaTiO_2.4_H_0.3_N_0.1_ – BaTiO_2.4_N_0.4_. Furthermore, Masuda et al. [40] have reported that even N_2_ can yield oxynitride BaTiO_2.5_N_0.2_ at 500 °C. Presumably, once the labile hydride anion leaves, the Ti cation becomes coordinatively unsaturated, and thus becomes active enough to react with N_2_ gas despite its triple N–N bond.

Apart from the thermal lability, an acid-base reaction involving hydride has also been invoked to yield new mixed-anion compounds. Masuda et al. reacted BaTiO_2.5_H_0.5_ with controlled amounts HF, to yield the mixed oxide-hydride-fluoride compound BaTiO_2.5_H_0.25_F_0.25_ [40]. The closely related compound BaTiO_2.9_F_0.1_ has so far been reported only by the use of high temperature and pressure (3 GPa, 1300 °C) [50]. Further F^–^/OH^–^ exchange is possible from here, as demonstrated by the preparation and characterization of BaTiO_2.4_D_0.3_(OD)_0.3_ from BaTiO_2.4_D_0.3_F_0.3_. This compound is an unusual case involving the co-existence of H^–^ and H^+^ (or in actuality, D^–^ and D^+^) within the same lattice, and has been made possible by the anion-exchange techniques starting from the labile oxyhydride precursor.

The two aforementioned reactions involve the direct exchange of H^–^ with other anions. However, even at higher temperatures where the hydride has already left, the remaining anion-deficient structure can be useful for further anion-exchange reactions. This has been demonstrated by the formation of EuTiO_2_N by Mikita et al. [41]. Here, EuTiO_2.82_H_0.18_ was first prepared. Treating with NH_3_ at 400 °C resulted in direct N^3–^/H^–^ exchange, to yield EuTiO_2.82_N_0.1_▢_0.06_. Further treatment at 800 °C resulted in the further oxygen to be removed, despite no hydride being present, to yield EuTiO_2_N. This contrasts with the reactivity of EuTiO_3_, which shows no reactivity with NH_3_ even at 600 °C. Treatment of EuTiO_3_ at 800 °C result in EuTiO_2.25_N_0.75_, but the composition EuTiO_2_N is not attained, unlike in the case when the oxyhydride was used as the starting material. This implies that the presence of the anionic vacancy during the reaction (2% in EuTiO_2.82_N_0.1_▢_0.06_) has a significant impact on the anion-exchange reaction.

## 5. Physical properties in oxyhydrides: transport and magnetism

### 5.1. Titanate oxyhydride perovskites

The alkali earth titanate oxyhydrides exhibit semiconducting to metallic character, depending on the hydride doping amount. Loosely pressed pellets of powder BaTiO_3–*x*_H_*x*_ (100 nm) exhibit semiconducting temperature dependences with a room temperature conductivity of 4 × 10^−4^ S/cm. This is probably not the inherent behavior, though, as single crystal films deposited by pulsed laser deposition on substrates yielded comprehensive results, showing metallic behavior at high doping levels.

Yajima et al. and Bouilly et al. have examined the conductivity of various titanate perovskite oxyhydride films [31,32]. SrTiO_2.75_H_0.25_, BaTiO_2.36_H_0.64_, and CaTiO_2.32_H_0.69_ films on (LaAlO_3_)_0.3_(SrAl_0.5_Ta_0.5_O_3_)_0.7_ (LSAT) substrates showed high conductivities at room temperature, ranging from 10^2^ to ~10^4^ S/cm, and detailed temperature-dependent studies showed metallic behavior. The SrTiO_2.75_H_0.25_ films are somewhat analogous to previously reported Sr(Ti_0.8_Nb_0.2_)O_3_ films [51], in terms of doping level, carrier concentration, and conductivity, with both systems essentially being heavily *n*-doped systems. For the barium titanate system, one may compare the BaTiO_2.36_H_0.34_ films to reduced BaTiO_3_. A key difference is that it is difficult to introduce a large number of oxygen vacancies in the perovskite structure due to structural instability, but extremely high doping levels can be achieved with hydride, as the anionic sites are always filled. The reduced BaTiO_3_ films, with very small carrier concentrations, were metallic, with several ferroelectric transitions resulting in anomalies on *ρ*–*T* curves [52]. However, the BaTiO_2.36_H_0.34_ samples exhibited no such anomalies. The dependence of electrical properties on hydride content for SrTiO_3–x_H_x_ and BaTiO_3–x_H_x_ films has also been examined. While the Sr films always exhibit metallicity the Ba films are semiconducting at low hydride amounts. The reason for this may be the same as for Nb-doped (Ba, Sr)TiO_3–δ_, where incoherent displacement of Ti from the octahedral center led to increased electron scattering [53].

Another titanate oxyhydride, EuTiO_2.7_H_0.3_ has been synthesized [30]. Thin film studies also show metallic conductivity for this sample due to 3*d* electrons of Ti, induced from the hydride reduction. The *A*-site europium is in the +2 oxidation state, and has ferromagnetic order (*T*_c_ = 12 K) from spins on Eu 4*f* orbitals being mediated by itinerant Ti 3*d* electrons, in contrast to EuTiO_3_ which is an antiferromagnetic insulator. The ferromagnetism based on the RKKY mechanism is also found in Eu_1–*x*_Ln_*x*_TiO_3_ and EuTi_1–*x*_Cr_*x*_O_3_, but here *T*_c_ is somewhat lower, implying the superiority of aliovalent hydride substitution at the anionic site in comparison with the cationic counterpart.

### 5.2. Vanadate oxyhydride perovskites and derivatives: SrVO_2_H, Sr_2_VO_3_H, Sr_3_V_2_O_5_H_2_

The electronic and magnetic structures of SrVO_2_H, and its related layered analogues, Sr_2_VO_3_H, and Sr_3_V_2_O_5_H_2_, (Figure 1(c), (f), and (g)) have been examined in detail [14,54,55]. These stoichiometric systems possess anion ordering, making them suitable for systematic studies. As shown in Figure 1(c), in the SrVO_2_H case, hydride occupies ‘apical’ sites (or *trans* octahedral VO_4_H_2_), whereas in the layered cases (Figure 1(f) and (g)), equatorial sites are occupied by hydride. Experimentally, neutron diffraction and muon spin relaxation shows that all three compounds have antiferromagnetic states. The Néel temperatures (*T*_N_) are 170 and 240 K for Sr_2_VO_3_H and Sr_3_V_2_O_5_H_2_ [15]. Surprisingly, the Néel temperature of SrVO_2_H is well above room temperature despite the two-dimensional structure. Sr_2_VO_3_H and Sr_3_V_2_O_5_H_2_ are regarded, respectively, as quasi one- and two-dimensional magnets. The nearly temperature-independent susceptibility and the absence of anomalies near at the ordering temperatures may reflect the low-dimensional feature of these compounds, with short-ranged magnetic correlations being developed well above *T*_N_ [15].

**Figure 5.**
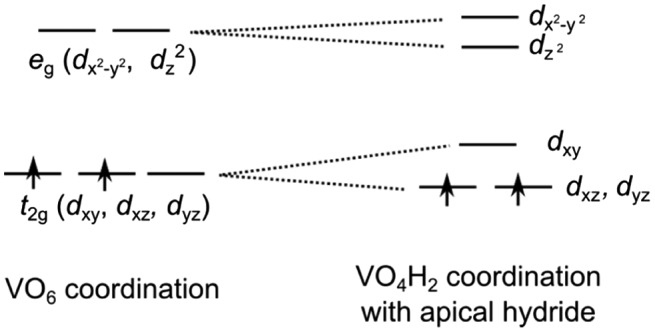
Molecular orbital diagram for SrVO_2_H.

The bonding and electronic structure in this system has been summarized by Romero et al. [15], Bang et al. [14] and Wei et al. [54], among others. A simple point is shown for SrVO_2_H in Figure 1(c). Here, H^–^ occupies the ‘apical’ site. For oxides such as SrVO_3_, the *t*_2g_ orbitals would remain triply degenerate and each form *π* bonds with O 2*p* orbitals (Figure 5). Additionally, H^–^ cannot form *π* bonds since it consists of only *s* orbitals. Hence, given the hydride in the ‘apical’ position, the *d*_xz_ and *d*_yz_ orbitals experience less repulsion and decrease in energy, splitting the *t*_2g_ state into a doubly degenerate *d*_xz_ and *d*_yz_ bands with a *d*_xy_ band above. The two *d* electrons partially fill the lower *d*_xz_ and *d*_yz_ bands; calculations predict the bands to have narrow dispersion, inducing a Mott transition to an insulator. This contrasts with the metallic SrVO_3_, with band valence photoelectron spectroscopy on thin film samples confirming this difference [55]. The experimentally observed antiferromagnetic ordering has also been predicted by spin-polarized band structure calculations [54].

Recently, there has been much work concerning electronic doping VO_2_ using various species. Doping VO_2_ results in metal-insulator transitions, and so far oxide vacancies [56] and protons (H^+^/e^–^, via H_2_ reduction) [57] have been introduced for *n*-doping to achieve alternate electronic states. Proton doping (via H_2_ reduction) has the advantage of not inducing disorder, but hydride is unique given its *s* character, and should therefore be a valuable addition to the various tools available for the modulation of electronic states, which have recently been garnering attention [58,59].

### 5.3. Cobalt oxyhydride perovskite derivatives: LaSrCoO_3_H_0.7_, Sr_3_Co_2_O_4.33_H_0.84_

While the non-stoichiometric nature of the cobalt oxyhydrides has limited electronic structural calculations and thus close examination of electronic structure, the cobaltates, starting with LaSrCoO_3_H_0.7_, were the first reported transition metal oxyhydrides [8]. As shown in Figure 1(a), the structure of LaSrCoO_3_H_0.7_ is based a Ruddlesden–Popper layered perovskite, but with hydride anions and vacancies preferentially occupying select equatorial sites to form an orthorhombic lattice. As in the case of the previously mentioned vanadates, antiferromagnetic order is observed at room temperature, signifying that strong covalent interactions through the Co-H-Co chains, together with the larger magnetic moments, contribute to the enhanced *T*_N_. Sr_3_Co_2_O_4.33_H_0.84_ (Figure 1(e)) is based on the *n* = 2 member of the Ruddlesden–Popper series [10]. While hydride has been incorporated, at this point oxygen removal is extensive, resulting in a mixture of tetrahedra and square pyramids. This extensive anion disorder and amount of vacancies is probably the reason for the lack of any observed magnetic order [10].

### 5.4. SrCrO_2_H

**Figure 6.**
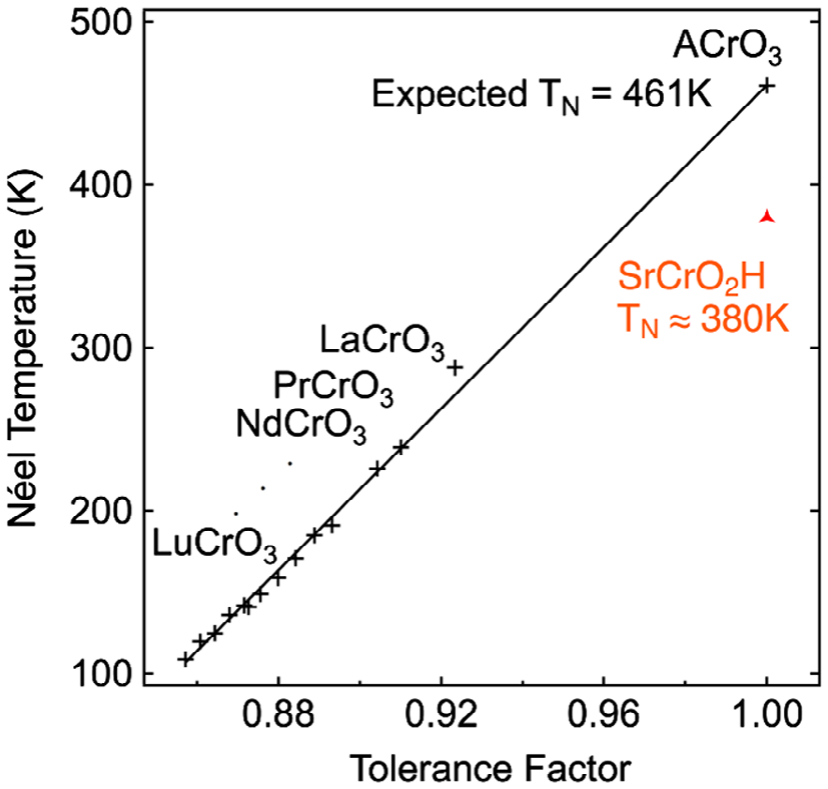
Change of Néel temperatures of various RECrO_3_ (RE = rare earth) phases with the tolerance factor.

The transition metal oxyhydride family was further expanded to chromates using high-pressure and high-temperature synthesis. SrCrO_2_H was prepared by heating powders of SrO, SrH_2_ and Cr_2_O_3_ at 5 GPa and 1000 °C [16]. The successful synthesis of this material probably benefits from the high stability of the Cr^3+^ cation. Trivalent chromium oxides do not reduce even under harsh reducing conditions, allowing the high-pressure/high-temperature and hydride-rich environment sustainable for this species. The structure is cubic and its stoichiometry could be determined using a combination of X-ray and neutron diffraction, as previously used for LaSrCoO_3_H_0.7_. The oxide and hydride anions do not order in this structure, thus explaining the cubic structure. The magnetic properties of SrCrO_2_H were studied by variable-temperature NPD, revealing a G-type antiferromagnetic order with a Néel temperature as high as 380 K. The *T*_N_ observed in this system is much higher than those of isovalent RECr^III^O_3_ (RE = rare earth) structures with the highest previously known value for LaCrO_3_ at *T*_N_ = 290 K. At a first glance, this behavior is strange as the octahedral environment in Cr^3+^ (*t*_2g_^3^) only allows π-type superexchange interactions (with the neighboring ligand) that cannot occur with the 1s orbital of the H^–^ anion. However, an hypothesis for this behavior is that the high symmetry of the SrCrO_2_H with a large Sr^2+^ cation (144 pm) gives a tolerance factor near 1; the cubic structure leads to ideal 180° Cr–O–Cr bond angles that allow strong superexchange antiferromagnetic interactions. In the case of oxide perovskites with smaller trivalent cations at the A site, distortions exist with tilting of the octahedra that leads to smaller *T*_*N*_. As shown in Figure 6, the Néel temperature of Cr^3+^ perovskites increase linearly with the tolerance factor but SrCrO_2_H has a slightly lower than expected Néel temperature.

Liu and coworkers investigated the electronic and magnetic properties of SrCrO_2_H using DFT calculations on a hypothetical ordered trans-coordinated model isostructural with the reported SrVO_2_H [60]. In their study, the authors speculated a possible secondary effect where the hydride’s lower electronegativity was partially responsible for the high *T*_N_. The lower electronegativity of hydride should lead to delocalized orbitals along the Cr–H bonds, which in turn reinforces Cr–O bonds and strengthens the superexchange interactions along Cr–O–Cr paths.

### 5.5. LaSrMnO_3.3_H_0.7_

The synthesis of LaSrMnO_3_H_0.7_ was achieved via high pressure and high temperature at 5 GPa and 1000 °C on using powders of La_2_O_3_, MnO, Mn_2_O_3_, SrH_2_ [17]. LaSrMnO_3.3_H_0.7_ crystallizes in the tetragonal *I*4/*mmm* space group with disorder of the oxide and hydride anions mainly in the equatorial plane (Figure 1(d)). This configuration confirms the trend observed also in cobaltate LaSrCoO_3_H_0.7_ and vanadates Sr_2_VO_3_H and Sr_3_V_2_O_5_H_2_. An important difference, however, lies in the lack of ordering of the two anions and therefore the observation of tetragonal symmetry rather than the orthorhombic symmetry found in the ordered Co and V systems. The anionic non-stoichiometry, determined by synchrotron X-ray and neutron diffraction, leads to a mixed valence Mn^2+^ and Mn^3+^ system with an average valence of Mn^2.3+^. While magnetic susceptibility measurements confirm this valence, they also exhibit magnetic behavior suggesting a spin glass transition at *T*_SG_ = 22 K. The absence of magnetic Bragg reflections in the low temperature neutron diffraction is consistent with this spin glass picture. The negatively large value of a Curie–Weiss temperature θ = –149 K suggests the strong magnetic interactions. A fairly large frustration factor of *f* ~ 6 (|θ|/*T*_SG_) was discussed in terms of competing magnetic interactions between neighboring Mn cations, i.e. ferromagnetic Mn^2+^–O–Mn^3+^ and antiferromagnetic Mn^3+^–O–Mn^3+^ and Mn^2+^–O–Mn^2+^.

## 6. Crystallographic site selectivity of hydride in layered systems

Total or partial anion ordering has been the topic of many studies for a long time in various mixed anion transition metal materials isolated so far, including oxyhalides and oxynitrides. Besides DFT first-principles calculations, energy calculations (the Madelung part of the lattice energy) and the bond valence sum method, basics Pauling’s electronegativity arguments have been evoked, whereby the more electronegative anion would be preferentially coordinated to the more electropositive cation, but without a total success in oxyfluorides, oxynitrides, or oxychlorides [61]. Another simple and general method for predicting the anion distribution not requiring the exact knowledge of the crystal structure has been proposed by Fuertes [62] according to the historic Pauling’s Second Crystal Rule (PSCR) published in 1929 [63] as one of the principles governing the structure of complex ionic crystals. It is obviously assumed each cation is coordinated to anions at the corner of a polyhedron and vice versa. Those principles have been deduced from empirical knowledge of known crystal structure at that time, as well as from stability considerations in terms of crystal energy. Their application scope was limited by Pauling himself [63] as follows: small cations with relatively large electric charges (+3 or +4 typically) and crystal radii lower than about 0.8 Å; large univalent or divalent anions (over 1.35 Å) *not too highly deformable* such as oxygen or fluorine anions. Furthermore the chemical bonds in the crystal should not be of the ‘extreme non-polar or shared electron pair type’, thus excluding copper compounds and ‘many other eighteen-shell atoms’. The PSCR or electrostatic valence principle [63] is based on the so-called strength *s* of the electrostatic valence bond (between one given cation and each adjacent anion) defined as $s=\frac{z}{\nu }$ where *z* is the formal charge of the cation and *ν* its coordination number (in anions). In a stable coordination structure, the formal charge –*ζ* of each anion (coordinated to *C* cations) approximately satisfies the following equation:

$$\left|\zeta \right|=\sum_{i=1}^{C}z_{i}/\nu_{i}=\sum_{i=1}^{C}s_{i}.$$

In materials (with anions of different charge) exhibiting chemically inequivalent anionic sites, for instance in lower than cubic symmetry layered perovskite structures, one can calculate according to the PSCR the charge –*ζ* of each anionic site that should be rather close to the charge of the anion actually occupying this site, allowing to predict the distribution of anions. Fuertes et al. [61,62] have shown this predictive method works well for many layered oxynitrides, oxyfluorides and oxychlorides (with the noteworthy exception of Nd_2_AlO_3_N [64], for which the difference between *ζ*_equatorial_ and *ζ*_apical_ calculated according to the PSCR, around 0.15, is very fine): the more charged anion would preferentially occupy the site showing the larger PSCR charge and conversely. Moreover for oxynitrides, oxyfluorides and one oxybromide of other structural types (e.g. antifluorite, baddeleyite, Th_3_P_4_, wurtzite …), PSCR appears as generally fulfilled though to a less extent for oxychlorides.

The discovery of LaSrMnO_3.3_H_0.7_ [17] by Tassel et al. has been naturally a motivation to confront this PSCR method with layered oxyhydride systems (including the previously published LaSrCoO_3_H_0.7_ [8], NdSrCoO_4–*x*_H_*y*_ [9], PrSrCoO_4–*x*_H_*y*_ [9] cobaltates or Sr_2_VO_4–*x*_H_*x*_ vanadates [14,15] in order to check its applicability range over this new series of mixed anion systems. The PSCR calculated anion formal charge at the equatorial site appears as systematically higher than at the apical site (see Table 1), leading to a predicted apical site preference for H^–^ while in all the K_2_NiF_4_ transition metal oxyhydrides hydride anions are located only on the equatorial site, except to be complete the case of Sr_2_VO_3.62_H_0.38_ and Sr_2_VO_2.99_H_1.01_ [14] though extremely low chemical occupancy of respectively 4 and 1.5% for H^–^ are suggested on the apical site (vs. 15 and 93%/5% on the equatorial site(s)). Hence it turns out the PSCR is not able to predict the anion ordering in transition metal oxyhydrides. However it should be stressed that the difference between PSCR *ζ*_equatorial_ and *ζ*_apical_ remains small for all the highlighted examples of Table 1, reaching at best 0.28.

**Table 1.** Formal charge of the apical and equatorial anionic site in known K_2_NiF_4_ transition metal oxyhydrides calculated according to the Pauling’s Second Crystal Rule (PSCR).

Oxyhydride	*ζ*_apical_	*ζ*_equatorial_
LaSrMnO_3.3_H_0.7_	–1.61	–1.89
LaSrCoO_3_H_0.7_	–1.67	–1.68
NdSrCoO_4−*x*_H_*y*_	–1.67	–1.68
PrSrCoO_4−*x*_H_*y*_	–1.67	–1.68
Sr_2_VO_4−*x*_H_*x*_	–1.77	–1.88

One singularity of hydride anion that could also explain the violation of the PSCR in oxyhydrides is its small Pauling’s electronegativity (2.1), as low as astatine, 2.2, the rare heaviest halogen, that may be too small for an electrostatic-based principle. It is worth noticing the lowest electronegative anion used by Fuertes et al. [61,62] to support the PSCR is bromine (*χ* = 2.96). Also hydride is highly polarizable and shows in crystals an ill-defined size ranging from 1.35 to 1.53 Å depending on the covalent to ionic bond character with a metal [65], property that strongly contradicts PSCR’s prerequisites underlined above in terms of non-deformability of anions.

Ordering/disordering of hydride within the equatorial plane of layered oxyhydrides has been controlled by Bouilly et al. [33] in epitaxial thin films through the use of different orientations of the LSAO (LaSrAlO_4_) substrate leading to two orientations (perpendicular or parallel) of the layer *c*-axis of the oxyhydride with respect to the substrate. CaH_2_-topochemical conversion of the *c*-axis and *b*-axis oriented LaSrCoO_4_ films (deposited by pulsed laser deposition on LSAO (0 0 1) and LSAO (1 0 0) substrates, respectively) leads to LaSrCoO_3_H_0.7_ oxyhydride films with tetragonal and orthorhombic symmetry, respectively. The latter and former cases correspond respectively to a crystallographically ordered and disordered hydride/oxide arrangement within the equatorial plane (see Figure 1(a) of [33]). In other words, the anionic order observed in bulk LaSrCoO_3_H_0.7_ [34] is recovered when the precursor oxide is deposited on the LSAO with *c*-axis within the film surface, while remarkably an original hydride/oxide disorder is created within the equatorial plane when the precursor oxide is deposited with a *c*-axis perpendicular to the film surface. These results demonstrate that strain engineering can lead to new materials with designed anion arrangement in mixed anion materials.

## 7. Summary and outlook

As discussed in this review, oxyhydrides have gone through rapid development over the past 10 years. We have summarized the various compounds and their characteristics in Table 2. Among the 3*d* transition metals, only Fe, Cu, and Zn oxyhydrides remain to be discovered. In terms of properties, hydride has certain characteristics unlike any other mixed anions. The 1*s* character gives it unusual bonding and hence magnetic/electronic implications, and may be useful in controlling the electronic properties of various materials with applications in devices. In terms of ionic conductivity, recently, reports on pure hydride ion conductivity for layered non-transition metal hydride perovskites have been reported [66], opening the doorway to hydride-based ionics. One can even imagine high-potential electrochemical cells based on the H^–^/H^+^ redox couple; such applications would not be possible without a wide range of hydride-based materials to choose from. We have also discussed the chemical lability of hydride, and this may serve as a useful approach to new hydrogenation catalysts based on (oxy-) hydrides. With these potential applications in devices, ionics, and catalysis, together with their fundamental scientific impact, it is evident that transition metal oxyhydrides will continue to gain prominence in the future.

**Table 2.** Summary of transition metal oxyhydride compounds.

Compound	Preparation*	Properties*	Anion arrangement*	Reference
LaSrCoO_3_H_0.7_	CaH_2_	I; AFM (*T*_N_ > RT); H^–^ diffusion	Partial order, eq.	[8,33,45]
NdSrCoO_3.1_H_0.80_, NdSrCoO_3.21_H_0.58_	CaH_2_	AFM (*T*_N_ = 410 K)	Partial order, eq.	[9]
		AFM (*T*_N_ = 375 K)		
PrSrCoO_3.16_H_0.68_	CaH_2_	AFM (*T*_N_ = 445 K)	Partial order, eq.	[9]
Sr_3_Co_2_O_4.33_H_0.84_	CaH_2_	PM	Partial order, eq.	[10]
(Ca, Ba, Sr)TiO_3–*x*_H_*x*_	CaH_2_	MS; PM	Random	[29,31,32]
EuTiO_3–*x*_H_*x*_	CaH_2_	M; FM from Eu moments	Random	[30]
		(*T*_c_ = 12 K)		
Ln_2_Ti_2_O_7−*x*_H_*x*_	CaH_2_	Electrocatalysis	Unknown (trace)	[37]
SrVO_2_H	CaH_2_	I; AFM (*T*_N_ > RT)	Ordered, apical	[15,55]
Sr_2_VO_3_H	CaH_2_	I; AFM (*T*_N_ = 170 K)	Ordered, equatorial	[15]
Sr_2_VO_3–*x*_H_*x*_	HP		Partial order, eq.	[14]
Sr_3_V_2_O_5_H_2_	CaH_2_	I; AFM (*T*_N_ = 240 K)	Ordered, equatorial	[15]
SrCrO_2_H	HP	I; AFM (*T*_N_ = 380 K)	Random	[16]
LaSrMnO_3.3_H_0.7_	HP	I; SG (*T*_SG_ = 22 K)	Partially ordered	[17]
BaScO_2_H	HP	I; local order (^1^H NMR, DFT)	Random	[13]

* CaH_2_ = reduction with CaH_2_, HP = high pressure, AFM = antiferromagnetism, FM = ferromagnetism, PM = paramagnetism, SG = spin glass, eq. = equatorial, I = insulating, M = metallic, S = semiconducting.

## Disclosure statement

No potential conflict of interest was reported by the authors.

## Funding

This work was supported by the CREST [No. JPMJCR1421] program from the Japan Science and Technology Agency (JST); and Grant-in-Aid for Scientific Research on Innovative Areas ‘Mixed anion’ [JP16H06439] from MEXT.

## References

[1] Norby T, Widerøe M, Glöckner R, et al. Hydrogen in oxides. Dalton Trans. 25;2004:3012–3018 10.1039/B403011G 15452624

[2] Brice JF, Moreau A Synthèse et conductivité anionique des hydruro-oxydes de lanthane de formule LaHO, LaH_1+2x_O_1-*x*_ et LaH_1+y_O_1-*x*_ (y < 2x). Ann Chim Fr. 1982;7:623–634.

[3] Malaman B, Brice JF Étude structurale de l’hydruro-oxyde LaHO par diffraction des rayons X et par diffraction des neutrons. J Solid State Chem. 1984;53:44–54. 10.1016/0022-4596(84)90226-3

[4] Rotella FJ, Flotow HE, Gruen DM, et al Deuterium site occupation in the oxygen‐stabilized η‐carbides Zr_3_V_3_OD_x_. I. Preparation and neutron powder diffraction. J Chem Phys. 1983;79:4522–4531. 10.1063/1.446339

[5] Clark NJ, Wu E Hydrogen absorption by M_5_X_3_ phase Zr-Al compounds. J Less Common Metals. 1988;142:145–154. 10.1016/0022-5088(88)90171-3

[6] Huang B, Corbett JD Ba_3_AlO_4_H: synthesis and structure of a new hydrogen-stabilized phase. J Solid State Chem. 1998;141:570–575. 10.1006/jssc.1998.8022

[7] Huang B, Corbett JD Ba_21_Ge_2_O_5_H_24_ and related phases. A corrected structure type and composition for a Zintl phase stabilized by hydrogen. Inorg Chem. 1998;37:1892–1899.

[8] Hayward MA, Cussen EJ, Claridge JB, et al The hydride anion in an extended transition metal oxide array: LaSrCoO_3_H_0.7_ . Science. 2002;295:1882–1884. 10.1126/science.1068321 11884751

[9] Bowman A, Claridge JB, Rosseinsky MJ Anion composition control and magnetic short- and long-range order in transition metal oxide hydrides. Chem Mater. 2006;18:3046–3056.

[10] Helps RM, Rees NH, Hayward MA Sr_3_Co_2_O_4.33_H_0.84_: an extended transition metal oxide-hydride. Inorg Chem. 2010;49:11062–11068. 21033678 10.1021/ic101613b

[11] Poulsen FW Speculations on the existence of hydride ions in proton conducting oxides. Solid State Ionics. 2001;145:387–397. 10.1016/S0167-2738(01)00935-3

[12] Kobayashi Y, Hernandez OJ, Sakaguchi T, et al An oxyhydride of BaTiO_3_ exhibiting hydride exchange and electronic conductivity. Nat Mater. 2012;11:507–511. 22504535 10.1038/nmat3302

[13] Goto Y, Tassel C, Noda Y, et al Pressure-stabilized cubic perovskite oxyhydride BaScO_2_H. Inorg Chem. 2017;56:4840–4846. 28398729 10.1021/acs.inorgchem.6b02834

[14] Bang J, Matsuishi S, Hiraka H, et al Hydrogen ordering and new polymorph of layered perovskite oxyhydrides: Sr₂VO_4−x_H_x_ . J Am Chem Soc. 2014;136:7221–7224. 10.1021/ja502277r 24802944

[15] Romero DF, Leach A, Möller JS, et al Strontium vanadium oxide-hydrides: “square-planar” two-electron phases. Angew Chem Int Ed Engl. 2014;53:7556–7559. 10.1002/anie.201403536 24962834

[16] Tassel C, Goto Y, Kuno Y, et al Direct synthesis of chromium perovskite oxyhydride with a high magnetic-transition temperature. Angew Chem Int Ed. 2014;53:10377–10380. 10.1002/anie.201405453 25115824

[17] Tassel C, Goto Y, Watabe D, et al High-pressure synthesis of manganese oxyhydride with partial anion order. Angew Chem Int Ed Engl. 2016;128:9819–9822. 10.1002/ange.201605123 27355695

[18] Hayward MA, Green MA, Rosseinsky MJ, et al Sodium hydride as a powerful reducing agent for topotactic oxide deintercalation: synthesis and characterization of the nickel(I) oxide LaNiO_2_ . J Am Chem Soc. 1999;121:8843–8854. 10.1021/ja991573i

[19] Seddon J, Suard E, Hayward MA Topotactic reduction of YBaCo_2_O_5_ and LaBaCo_2_O_5_: square-planar Co(I) in an extended oxide. J Am Chem Soc. 2010;132:2802–2810. 10.1021/ja910103d 20131833

[20] Dixon E, Hadermann J, Ramos S, et al Mn(I) in an extended oxide: the synthesis and characterization of La_1−x_Ca_x_MnO_2+δ_ (0.6 ≤ *x* ≤ 1). J Am Chem Soc. 2011;133:18397–18405. 10.1021/ja207616c 21999167

[21] Romero FD, Coyle L, Hayward MA Structure and magnetism of Sr_3_Co_2_O_4_Cl_2_ an electronically driven lattice distortion in an oxychloride containing square planar Co^II^ centers. J Am Chem Soc. 2012;134:15946–15952. 10.1021/ja306683y 22931346

[22] Tsujimoto Y, Tassel C, Hayashi N, et al Infinite-layer iron oxide with a square-planar coordination. Nature. 2007;450:1062–1065. 10.1038/nature06382 18075589

[23] Tassel C, Pruneda JM, Hayashi N, et al CaFeO_2_: a new type of layered structure with iron in a distorted square planar coordination. J Am Chem Soc. 2009;131:221–229. 10.1021/ja8072269 19128179

[24] Tassel C, Watanabe T, Tsujimoto Y, et al Stability of the infinite layer structure with iron square planar coordination. J Am Chem Soc. 2008;130:3764–3765. 10.1021/ja800415d 18314991

[25] Yamamoto T, Li Z, Tassel C, et al Synthesis and thermal stability of the solid solution AFeO_2_ (A = Ba, Sr, Ca). Inorg Chem. 2010;49:5957–5962. 20509630 10.1021/ic100452m

[26] Seinberg L, Yamamoto T, Tassel C, et al. Fe-site substitution effect on the structural and magnetic properties in SrFeO_2_ . Inorg Chem. 2011;50:3988–3995. 21452805 10.1021/ic102467u

[27] Poltavets VV, Lokshin KA, Dikmen S, et al La_3_Ni_2_O_6_: a new double T’-type nickelate with infinite Ni^1+/2+^O_2_ layers. J Am Chem Soc. 2006;128:9050–9051. 10.1021/ja063031o 16834375

[28] Hadermann J, Abakumov AM, Adkin JJ, et al Topotactic reduction as a route to new close-packed anion deficient perovskites: structure and magnetism of 4H-BaMnO_2+*x*_ . J Am Chem Soc. 2009;131:10598–10604. 10.1021/ja903216d 19722633

[29] Sakaguchi T, Kobayashi Y, Yajima T, et al Oxyhydrides of (Ca, Sr, Ba)TiO_3_ perovskite solid solutions. Inorg Chem. 2012;51:11371–11376. 23082857 10.1021/ic300859n

[30] Yamamoto T, Yoshii R, Bouilly G, et al An antiferro-to-ferromagnetic transition in EuTiO_3–x_H_x_ induced by hydride substitution. Inorg Chem. 2015;54:1501–1507. 25594721 10.1021/ic502486e

[31] Yajima T, Kitada A, Kobayashi Y, et al Epitaxial thin films of ATiO_3–x_H_x_ (A = Ba, Sr, Ca) with metallic conductivity. J Am Chem Soc. 2012;134:8782–8785. 10.1021/ja302465c 22563869

[32] Bouilly G, Yajima T, Terashima T, et al Electrical properties of epitaxial thin films of oxyhydrides ATiO_3–*x*_H_*x*_ (A= Ba and Sr). Chem Mater. 2015;27:6354–6359. 10.1021/acs.chemmater.5b02374

[33] Bouilly G, Yajima T, Terashima T, et al Substrate-induced anion rearrangement in epitaxial thin films of LaSrCoO_4−x_H_x_ . CrystEngComm. 2014;16:9669–9674. 10.1039/C4CE01268B

[34] Hayashi K, Matsuishi S, Kamiya T, et al Light-induced conversion of an insulating refractory oxide into a persistent electronic conductor. Nature. 2002;419:462–465. 10.1038/nature01053 12368851

[35] Hayashi K Heavy doping of H^–^ ion in 12CaO 7Al_2_O_3_ . J Solid State Chem. 2011;184:1428–1432. 10.1016/j.jssc.2011.04.008

[36] Kitada A, Kasahara S, Terashima T, et al Highly reduced anatase TiO_2−δ_ thin films obtained via low-temperature reduction. Appl Phys. Express. 2011;4:035801 10.1143/APEX.4.035801

[37] Pussacq T, Kabbour H, Colis S, et al Reduction of Ln_2_Ti_2_O_7_ layered perovskites: a survey of the anionic lattice, electronic features, and potentials. Chem Mater. 2017;29:1047–1057.

[38] Kobayashi Y, Li Z, Hirai K, et al. Gas phase contributions to topochemical hydride reduction reactions. J Solid State Chem. 2013;207:190–193. 10.1016/j.jssc.2013.09.006

[39] Hayashi K, Sushko PV, Hashimoto Y, et al Hydride ions in oxide hosts hidden by hydroxide ions. Nat Commun. 2014;5:1–8. 10.1038/ncomms4515 PMC3973043 24662678

[40] Masuda N, Kobayashi Y, Hernandez O Hydride in BaTiO_2.5_H_0.5_: a labile ligand in solid state chemistry. J Am Chem Soc. 2015;137:15315–15321. 10.1021/jacs.5b10255 26575595

[41] Mikita R, Aharen T, Yamamoto T, et al Topochemical nitridation with anion vacancy-assisted N^3–^-/O^2–^-exchange. J Am Chem Soc. 2016;138:3211–3217. 10.1021/jacs.6b00088 26855196

[42] Izumi F, Momma K 2011 Three-dimensional visualization of electron-and nuclear-density distributions in inorganic materials by MEM-based technology. In: IOP Conference Series: Materials Science and Engineering. Vol. 18 IOP Publishing p. 022001 10.1088/1757-899X/18/2/022001

[43] Yoshizumi T, Kobayashi Y, Kageyama H, et al Simultaneous quantification of hydride ions and electrons incorporated in 12CaO 7Al_2_O_3_ cages by deuterium-labeled volumetric analysis. J Phys Chem C. 2012;116:8747–8752. 10.1021/jp2106742

[44] Castro FJ, Meyer G Thermal desorption spectroscopy (TDS) method for hydrogen desorption characterization (I): theoretical aspects. J Alloys Compd. 2002;330–332:59–63. 10.1016/S0925-8388(01)01625-5

[45] Bridges CA, Fernandez-Alonso F, Goff JP, et al Observation of hydride mobility in the transition-metal oxide hydride LaSrCoO_3_H_0.7_ . Adv Mater. 2006;18:3304–3308.

[46] Lu X, Cheung NW, Strathman MD, et al Hydrogen induced silicon surface layer cleavage. Appl Phys Lett. 1997;71:1804–1806.

[47] Sandim HRZ, Morante BV, Suzuki PA Kinetics of thermal decomposition of titanium hydride powder using *in situ* high-temperature X-ray diffraction (HTXRD). Mater Res. 2005;8:293–297.

[48] Liu H, He P, Feng JC, et al Kinetic study on nonisothermal dehydrogenation of TiH_2_ powders. Int J Hydrogen Energy. 2009;34:3018–3025. 10.1016/j.ijhydene.2009.01.095

[49] Yajima T, Takeiri F, Aidzu K, et al A labile hydride strategy for the synthesis of heavily nitridized BaTiO_3_ . Nat Chem. 2015;7:1017–1023. 26587718 10.1038/nchem.2370

[50] Endo T, Kobayashi T, Sato T, et al High pressure synthesis and electrical properties of BaTiO_3–x_F_x_ . J Mater Sci. 1990;25:619–623. 10.1007/BF00714085

[51] Ohta H, Sugiura K, Koumoto K Recent progress in oxide thermoelectric materials: p-type Ca_3_Co_4_O_9_ and n-type SrTiO_3_ . Inorg Chem. 2008;47:8429–8436. 18821809 10.1021/ic800644x

[52] Kolodiazhnyi T Insulator-metal transition and anomalous sign reversal of the dominant charge carriers in perovskite BaTiO_3 − δ_ . Phys Rev B: Condens Matter Mater Phys. 2008;78:045107 10.1103/PhysRevB.78.045107

[53] Page K, Kolodiazhnyi T, Proffen T, et al Local structural origins of the distinct electronic properties of Nb-substituted SrTiO_3_ and BaTiO_3_ . Phys Rev Lett. 2008;101:205502 10.1103/PhysRevLett.101.205502 19113352

[54] Wei Y, Gui H, Li X, et al The effect of hydrogen ordering on the electronic and magnetic properties of the strontium vanadium oxyhydride. J Phys Condens Matter. 2015;27:206001 10.1088/0953-8984/27/20/206001 25950614

[55] Katayama T, Chikamatsu A, Yamada K, et al Epitaxial growth and electronic structure of oxyhydride SrVO_2_H thin films. J Appl Phys. 2016;120:085305 10.1063/1.4961446

[56] Zhang Z, Zuo F, Wan C, et al. Evolution of metallicity in vanadium dioxide by creation of oxygen vacancies. Phys Rev Appl. 2017;7:034008.

[57] Yoon H, Choi M, Lim T-W, et al Reversible phase modulation and hydrogen storage in multivalent VO_2_ epitaxial thin films. Nat Mater. 2016;15:1113–1119. 27400385 10.1038/nmat4692

[58] Shi J, Zhou Y, Ramanathan S Colossal resistance switching and band gap modulation in a perovskite nickelate by electron doping. Nat Commun. 2014;5:1–9. 10.1038/ncomms5860 25181992

[59] Lu N, Zhang P, Zhang Q, et al Electric-field control of tri-state phase transformation with a selective dual-ion switch. Nature. 2017;546:124–128. 10.1038/nature22389 28569818

[60] Liu K, Hou Y, Gong X, et al Orbital delocalization and enhancement of magnetic interactions in perovskite oxyhydrides. Sci Rep. 2016;6:1–7. 26804825 10.1038/srep19653 PMC4726173

[61] Tobías G, Beltrán-Porter D, Lebedev OI, et al Anion ordering and defect structure in Ruddlesden-Popper strontium niobium oxynitrides. Inorg Chem. 2004;43:8010–8017. 15578839 10.1021/ic049236k

[62] Fuertes A Prediction of anion distributions using Pauling’s second rule. Inorg Chem. 2006;45:9640–9642. 17112257 10.1021/ic061640r

[63] Pauling L The principles determining the structure of complex ionic crystals. J Am Chem Soc. 1929;51:1010–1026. 10.1021/ja01379a006

[64] Marchand R, Pastuszak R, Laurent Y Structure cristalline de Nd_2_AlO_3_N. Détermination de L’ordre oxygène-azote par diffraction de neutrons. Rev Chim. 1982;19:684–689.

[65] Morris DFC, Reed GL Pauling L.Crystal radius of the hydride ion. J Inorg Nucl Chem. 1965;27:1715–1717. 10.1016/0022-1902(65)80037-9

[66] Kobayashi G, Hinuma Y, Matsuoka S, et al Pure H^–^ conduction in oxyhydrides. Science. 2016;351:1314–1317. 10.1126/science.aac9185 26989251

